# Assessment of nutritional knowledge in female athletes susceptible to the Female Athlete Triad syndrome

**DOI:** 10.1186/1745-6673-2-10

**Published:** 2007-09-27

**Authors:** Philippa Raymond-Barker, Andrea Petroczi, Eleanor Quested

**Affiliations:** 1 School of Sport, Health and Exercise Sciences, University of Wales, Bangor, UK; 2 School of Life Sciences, Kingston University, Kingston upon Thames, UK; 3 School of Sport and Exercise Sciences, The University of Birmingham, Edgbaston, UK

## Abstract

**Background:**

The study aimed to i) assess nutritional knowledge in female athletes susceptible to the Female Athlete Triad (FAT) syndrome and to compare with controls; and ii) to compare nutritional knowledge of those who were classified as being 'at risk' for developing FAT syndrome and those who are 'not at risk'.

**Methods:**

In this study, participants completed General Nutritional Knowledge Questionnaire (GNKQ), the Eating Attitude Test (EAT-26) and survey measures of training/physical activity, menstrual and skeletal injury history. The sample consisted of 48 regional endurance athletes, 11 trampoline gymnasts and 32 untrained controls. Based on proxy measures for the FAT components, participants were classified being 'at risk' or 'not at risk' and nutrition knowledge scores were compared for the two groups. Formal education related to nutrition was considered.

**Results:**

A considerably higher percentage of athletes were classified 'at risk' of menstrual dysfunction than controls (28.8% and 9.4%, respectively) and a higher percentage scored at or above the cutoff value of 20 on the EAT-26 test among athletes than controls (10.2% and 3.1%, respectively). 8.5% of athletes were classified 'at risk' for bone mineral density in contrast to none from the control group. Nutrition knowledge and eating attitude appeared to be independent for both athletes and controls. GNKQ scores of athletes were higher than controls but the differences between the knowledge of 'at risk' and 'not at risk' athletes and controls were inconsequential. Formal education in nutrition or closely related subjects does not have an influence on nutrition knowledge or on being classified as 'at risk' or 'not at risk'.

**Conclusion:**

The lack of difference in nutrition knowledge between 'at risk' and 'not at risk' athletes suggests that lack of information is not accountable for restricted eating associated with the Female Athlete Triad.

## 

The dramatic increase in the number of women participating in sport and exercise has, for most, contributed to improved physical fitness, significant health benefits and consequently enhanced overall well-being [1]. However for some female athletes driven to excel, serious commitment to their chosen sport may increase the risk of developing a syndrome known as the 'Female Athlete Triad' [2-4]. The term 'Female Athlete Triad', was first coined in 1992 by the American College of Sports Medicine in response to several studies concluding that a number of female athletes suffer from the inter-related symptoms of disordered eating, amenorrhea and osteoporosis [2-4]. Alone, each disorder is of significant medical concern, but when all three components are present, the effects are synergistic and greater potential for serious negative impact on health develops [1]. Those competing in sports where low body weight is a prerequisite may be at increased risk [1], thus research has predominantly focused on adolescents or elite athletes involved in either endurance sports, such as running and cycling, or aesthetic type sports, such as ballet and gymnastics. The seriousness of this problem has been highlighted in the recent position stand of the International Olympic Committee Medical Commission [5].

A worrisome misconception among athletes and coaches is that cessation of menstruation occurs when body fat levels become optimal for any given sport, signifying appropriate training volume and intensity. Thus many athletes and coaches view unrealistically low body weight key to superior athletic performance. This leads to restricted diet and dieting behaviour is often considered the initiating factor of the Female Athlete Triad [6,7]. Restrictive eating behaviour, combined with excessive energy expenditure, leads to decreased body weight [2]. As well as weight loss, significant caloric restriction reduces metabolic rate and causes changes to the cardiovascular, muscular skeletal, thermoregulatory and endocrine systems. In some cases, menstrual abnormalities can be explained by low estrogen levels caused by a deficit in energy intake and expenditure due to restrictive eating behaviour or excessive training. Therefore, the absence of the menstrual cycle may be an energy-conserving strategy to protect more important biological and reproductive processes. Cessation of menstruation removes the protective effects of estrogens on bone, making women more vulnerable to calcium loss with concomitant decrease in bone mineral density [8]. As menstrual dysfunction is decidedly easier to recognise and diagnose than disordered eating or bone mineral density, it is often regarded as the 'red flag' for the Triad. The connection between menstrual irregularity had significantly higher mean scores on eating disorder questionnaires has been established [9].

Nutrition, the cornerstone of the Triad, has been perceived as a key component in preventing female specific health problems [10-12]. The conflicting argument is whether athletes' eating behaviour is influenced by nutrition knowledge [12] or whether in spite of this, an element of personal choice is the dominant factor [11]. This choice factor has been labelled 'cognitive dietary restraint' (CDR) and refers to the conscious efforts to limit food intake in order to maintain or achieve a desired body weight [13].

For example, a relatively early study [14] examined the relationship between disordered eating using the Eating Attitudes Test-26 (EAT-26) and nutrition knowledge and concluded that the level of nutrition knowledge attained by an athlete has a positive influence on eating behaviour. The link between nutrition knowledge and attitude was confirmed by showing that a relationship exists between nutrition knowledge and predisposition toward dietary restraint [13]. On the contrary, Packman and Kirk [15] suggested that nutrition knowledge is not an entirely independent factor determining dietary behaviour.

Zawila and colleagues [12] concluded that the female athlete appears to lack knowledge or else fails to comply with recommendations for other unknown reasons. This study suggests that further research is necessary to examine the relationship between the nutrition knowledge of athletes and the Triad components, in order to isolate possible reasons for restrictive eating behaviour for subsequent research in this field.

The aims of this study were to: i) assess the nutrition knowledge of athletes and controls, and ii) investigate whether there is a significant difference in mean nutrition knowledge scores of those athletes classified as 'at risk' and 'not at risk'. It was hypothesized that the levels of nutrition knowledge in 'at risk' and 'not at risk' populations do not differ significantly suggesting that nutrition knowledge (or lack of) is independent of the Triad syndrome.

## Methods

### Participants

Qualifying criteria for the athletic sample population were; i) female endurance athlete (i.e. runner, cyclist) or gymnast over 18 years of age [16], ii) competitive involvement in the previous or coming year, and iii) training for ≥ 5 hr/wk^-1^, considered frequent training [17]. As previous research showed that participation in certain sports (e.g. gymastics, running) increases the risk of the Triad [18,19], athletes from sport where leanness is considered to be advantageous were recruited via contact with local clubs. Respondents representing the normal population were randomly selected from personal and university email lists. The final sample was comprised of selected respondents from the respondent pool for both athletes and controls. Exclusion criteria for self-selecting candidates in both categories included pregnancy or severe injury that had prevented the candidate from physical activity for more than 3 months. Criteria for inclusion in the control sample were: i) age > 18, and ii) non-athlete. Questionnaires were administered to 88 athletes and 62 non-athletes. The response rate was 67% and 52% respectively. Participation in the study was voluntary and participants gave implied consent by returning the questionnaire. Participants were offered feedback if they wished to receive it, otherwise the questionnaire was anonymous. The final sample consisted of 59 athletes and 32 controls. Participants' characteristics are described in details in the results section.

### Assessment tool

Advice was sought regarding content validity of the complete questionnaire from a number of professionals, including a general practitioner, a gynaecologist, a sports dietician and physiologist. This was particularly important for the proxy measures for being 'at risk' for menstrual dysfunction and osteoporosis. A pilot study was performed amongst female sports science students (n = 6) providing feedback on content, format, understanding and ease of use and piloted once again with the same respondents two weeks later. The final survey packet contained the following tests:

i) Disordered eating was assessed with the EAT-26 [20]. The EAT-26 is a shortened version of the original EAT-40 scale [21] and published by Garner and colleagues as an economic and objective measure of the symptoms of anorexia nervosa [20]. The scale consists of 26 items tapping into three eating problems: dieting; bulimia and food preoccupation; and oral control. Statements are rated on a 5-point scale ranging from *never *through *rarely, sometimes, often, usually *to *always*. Answers marked as *never*, *rarely *and *sometimes *carry zero points whilst often = 1, usually = 2 and always = 3 points, except the last item which is reverse-coded. Respondents scoring ≥ 20 were considered 'at risk' [20]. In isolation, the scale does not yield a specific diagnosis of an eating disorder, however it is consistently used in a two-step diagnostic process as an effective screening instrument and has been found to be effective with clinical and sub-clinical populations. In addition, respondents were asked if they had ever been clinically diagnosed and/or treated for an eating disorder.

ii) Risk for menstrual dysfunction was assessed using an adaptation of the screening questions routinely used in the 'Eating, Sports and Health in Females' project of the Better Eating Safer Training Research Study (B.E.S.T.) research series [22]. Age of menarche, frequency and regularity of menstrual cycles, training associated changes in cycle regularity, both past and present, and oral contraceptive use were all established. Additionally, respondents were asked if they had ever been diagnosed with primary amenorrhea (lack of menarche), secondary amenorrhea (absence of more than 3 periods) or oligomenorrhea (irregular periods). All questions were allocated a red or amber 'flag' indicating the presence of a proxy for menstrual dysfunction. Scores above 1.5 were considered 'at risk'.

iii) Skeletal injury history was used in order to gauge bone mineral density (BMD) with questions concerning the type and frequency of skeletal injuries sustained during the respondent's athletic career. For the control population, injuries sustained since puberty were recalled. Questions were modified from those used in the B.E.S.T study [22]. Respondents were also asked if they had ever been clinically diagnosed with low bone mineral density or osteoporosis. Injury frequency exceeding one occurrence during competitive training and self reported osteoporosis/low bone mineral density qualified the respondent 'at risk'. There was no relationship between 'at risk' category for menstrual dysfunction and the use of oral contraceptives for athletes (χ^2 ^= 0.565, *p *= .452) or controls (χ^2 ^= 0.007, *p *= .935).

iv) Nutrition knowledge was examined using the General Nutrition Knowledge Questionnaire (GNKQ) [23]. The questionnaire is comprised of a total of 110 Yes/No and multiple choice questions in 4 sections, i) dietary recommendations, ii) sources of food/nutrients, iii) choosing everyday foods, iv) diet disease relationships and also asks respondents whether or not they have a degree in nutrition or related subjects. Each knowledge item carries one point for a correct answer. The total composite score from each knowldge section is used in the statistical analysis. Sample questions are shown in Table 1.

**Table 1:** Sample Questions from the General Nutrition Knowledge Questionnaire

Section	Sample questions
Expert advice	What version of diary foods do experts say people should eat? (*tick one*)
	(a) full fat
	(b) lower fat
	(c) mixture of full fat and lower fat
	(d) neither, diary foods should be cut out
	(e) not sure

	How many servings of fruit and vegetables a day do you think experts are advising people to eat? (One serving could be, for example, an apple or a handful of chopped carrots)

Food groups	There is more protein in a glass of milk than in a glass of skimmed milk.
	(a) agree
	(b) disagree
	(c) not sure

	Which do you think is higher in calories: butter or regular margarine? (*tick one*)
	(a) butter
	(b) regular margarine
	(c) both the same
	(d) not sure

Choosing foods	If a person wanted to reduce the amount of fat in their diet, which would be the best choice? (*tick one*)
	(a) steak, grilled
	(b) sausages, grilled
	(c) turkey, grilled
	(d) pork chop, grilled

	Which would be the best choice for a low fat, high fibre snack? (*tick one*)
	(a) grilled chicken
	(b) cheese on wholemeal toast
	(c) beans on wholemeal toast
	(d) quiche

Health problems	Are you aware of any major health problems or diseases that are related to the amount of fat people eat?
	(a) yes
	() no
	(c)not sure
	If yes, what diseases or health problems do you think are related to fat?

	Which one of these is more likely to raise people's blood cholesterol level? (tick one)
	(a) antioxidants
	(b) polyunsaturated fats
	(c) saturated fats
	(d) cholesterol in the diet
	(e) nor sure

v) Demographic information included age, sport, past and present sport/exercise activity. Total training for the athletes was defined as the total number of hours training per week. For the control population, amount of physical activity was defined as the total number of hours per week including recreational sports and daily activities such as walking. As a control measure, respondents were asked whether they had formal education in nutrition or closely related subjects.

### Statistical analyses

All analyses were performed using SPSS software, version 14.0. Results are expressed as mean value and standard deviation or number of respondents and percentage. Reliability for EAT-26 was established using Cronbach alpha and Kuder-Richardson 21 formula (KR-21) for the General Nutrition Knowledge Questionnaire. Pearson product moment correlation coefficients (*r*) were used to test for a significant relationship between nutritional knowledge and eating attitude. Chi-square statistics were used to test independence of research variables (i.e. menstrual dysfunction or being 'at risk') and possible confounding variables (i.e. using oral contraceptive, formal education in nutrition or type of sport). Group differences in quantitative measures (i.e. weight, height, knowledge scores) were tested by *t*-test procedure where appropriate. Due to the overall small sample and unequal sample sizes, comparisons between athletes and controls were performed using Mann-Whitney *U *and Kruskal-Wallis *H*. Among other assumptions, parametric methods assume normality or sample size > 100 in each group to be compared. The 'at risk' groups, owing to the nature of the problem, are usually a magnitude smaller than their 'not at risk' counterparts. Nonparametric statistical methods relax these fundamental assumptions of the parametric comparison, thus allows researchers to test statistical significance in these special cases. Non-parametric equivalent tests are also the most appropriate when the sample sizes are small [24]. Differences were considered statistically significant for *p *< .05.

## Results

### Characteristics of the participants

The athlete sample (n = 59) consisted of 48 endurance athletes (81%) and 11 trampoline gymnasts (19%). Table 2 summarises the anthropometric data for each sport group and controls. No significant difference was found in age between all athletes and controls (*t *= 1.073, *p *= .286; *d *= .240) or height (*t *= 1.719, *p *= .089). Weight (*t *= -2.531, *p *= .013) and BMI (*t *= 2.453, *p *= .016) were significantly lower for the athletic population compared to the controls.

**Table 2:** Anthropometric Data and Training Volume of Athletes and Controls (Mean and Standard Deviation in parentheses)

Group	n	Mean age (years)	Mean weight (kg)	Mean height (cm)	Mean BMI (kg/m^-2^)	Training (hr/w)
Runners	20	36.50 (9.03)	58.65 (8.20)	162.70 (7.41)	22.15 (2.71)	9.85 (4.17)
Cyclists	4	37.20 (6.26)	59.00 (5.48)	164.80 (5.72)	21.71 (1.66)	9.00 (4.18)
Triathletes	24	37.04 (7.58)	58.71 (6.06)	165.50 (5.35)	21.45 (2.06)	8.04 (2.16)
All endurance athletes	48	37.00 (7.95)	58.90 (6.79)	164.38 (6.36)	21.81 (2.30)	8.96 (6.36)
Trampoline gymnasts	11	20.27 (1.62)	50.36 (26.34)	151.73 (50.71)	18.01 (9.22)	7.82 (2.36)
All athletes	59	33.88 (9.74)	57.31 (12.97)	162.02 ± (22.38)	21.10 (4.60)	8.75 (3.20)
Controls	32	31.69 (8.47)	65.50 (17.59)	169.09 (8.49)	23.57 (4.43)	3.75 (2.75)

For classification, the 'at risk' criteria were adapted from literature precedents [23] as follows: i) disordered eating was indicated by EAT-26 score ≥ 20, ii) menstrual dysfunction (changing, irregular or missed periods for more than 3 months) score > 1.5 or being diagnosed with amenorrhea or oligomenorrhea; and iii) the indicator for problems with bone mineral density was having more than one incidents (i.e. stress fracture, broken bone, compression fracture, curving of spine or humpback) or being diagnosed with low bone mineral density or osteoporosis. In addition, BMI < 18.5 is also considered a sign for being 'at risk' for the Triad. Internal reliability for EAT-26 was well above the customary cutoff value for athletes (α = .899 > .7) and was acceptable for controls (α = .760 > .7).

Owing to the special characteristics of the sport, trampoline gymnasts differed significantly from both controls and endurance athletes in age (*F *= 21.944, *p *< .001), training hours per week (*F *= 28.949, *p *< .001), weight (*F *= 4.811, *p *= .01), height (*F *= 3.642, *p *= .030) but not in BMI (*H *= 4.857, *p *= .088). However, the type of sport was unrelated to the prevalence of risks for disordered eating (χ^2 ^= .899, *p *= .343), menstrual dysfunction (χ^2 ^= .016, *p *= .900) and osteoporosis (χ^2 ^= 1.643, *p *= .200), thus there was no need to treat endurance athletes and gymnasts separately for the purpose of this investigation. Having formal education in nutrition or related subjects did not have an influence on nutrition knowledge (*U *= 147.0, *p *= .104) or on having symptoms for the Female Athlete Triad (χ^2 ^= .925, *p *= .336).

Significant differences between athletes and controls were only observed for overall risks (*U *= 620.00, *p *= .007) and for menstrual dysfunction (*U *= 699.00, *p *= .028) as an individual component of the Triad. No difference was found in risks for osteoporosis (*U *= 789.00, *p *= .101) or disordered eating (χ^2 ^= 1.328, *p *= .249). The difference in mean EAT-26 scores also proved to be insignificant (*t *= 1.88, *p *= .062) between athletes and controls.

**Figure 1:**
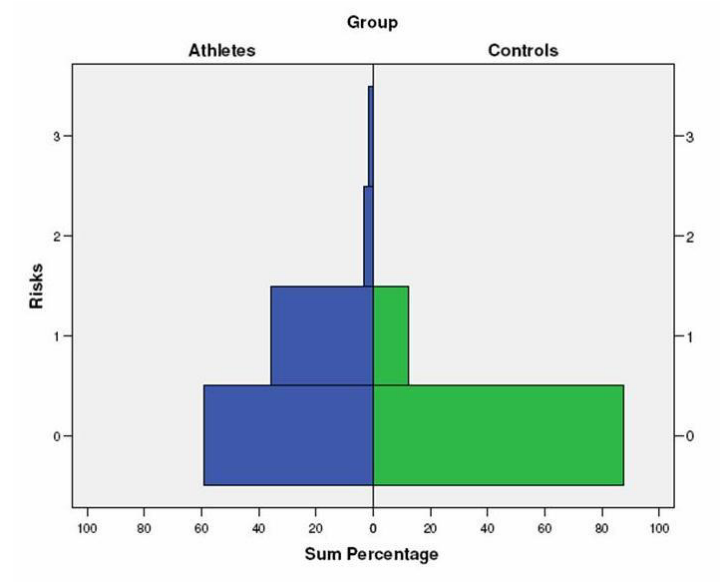
Proportion of athletes and controls in one, two or all component of the Female Athlete Triad (0 = no risk, 1 = 'at risk' for menstrual dysfunction, 2 = 'at risk' for disordered eating, 3 = 'at risk' for osteoporosis).

**Figure 2:**
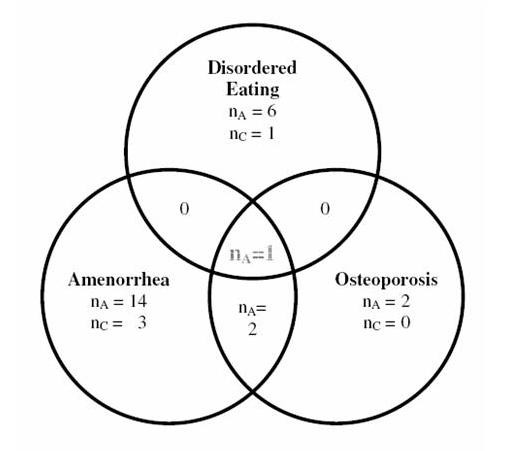
Number of cases in each component of the Female Athlete Triad for athletes (n_A _= 59) and controls (n_C _= 32).

Figure 1 and 2 show that a greater proportion of athletes are presently experiencing or have in the past experienced components of the Triad. Figure 1 clearly illustrates that more athletes are 'at risk' of one, two or all components of the Triad than controls. The highest percentage of being 'at risk' was observed in menstrual dysfunction with 28.8% among athletes compared to 9.4% among the controls. Based on the EAT-26 test scores, 10.17% of athletes were classified 'at risk' for disordered eating (3.12% among controls). Indicators for being 'at risk' for osteoporosis placed 8.47% of the athletes into the 'at risk' category and there were no controls in this group.

Being 'at risk' in more than one component only occurred among athletes. While no athletes were classified 'at risk' for a combination of disordered eating and low bone mineral density or for a combination of disordered eating and menstrual dysfunction; two athletes were 'at risk' of low bone mineral density alongside menstrual dysfunction (Figure 2). One athlete was considered 'at risk' of all 3 aspects of the triad. These results are congruent with the literature [25]. None of the control respondents were 'at risk' of all three components. None appeared to be 'at risk' of low bone mineral density and disordered eating or menstrual dysfunction and disordered eating in combination. For further analysis, overall 'at risk' was operationally defined as being 'at risk' in at least one component of the Triad.

### Data characteristics

Internal reliability of the General Nutritional Knowledge Questionnaire was excellent for both athletes (*KR-21 *= .893) and controls (*KR-21 *= .887). Of the 91 respondents, 10 athletes and 8 controls indicated having a degree in nutrition or in related subjects but the proportions of respondents with a nutrition degree did not show any specific pattern (χ^2 ^= 0.706, *p *= .401). There was no interaction observed between having a degree and athletic status in nutrition knowledge (*F *= 0.563, *p *= .455) thus the main effects can be interpreted independently. Total mean scores were 81.46 ± 12.13 and 75.31 ± 12.93 for athletes and controls respectively and the difference was statistically significant (*t *= 2.254, *p *= .027). Similarly, those with a degree scored significantly higher on the General Nutritional Knowledge Questionnaire than those without a degree (87.00 ± 7.42 and 79.91 ± 12.69, respectively; *t *= 2.594, *p *= .011).

**Figure 3:**
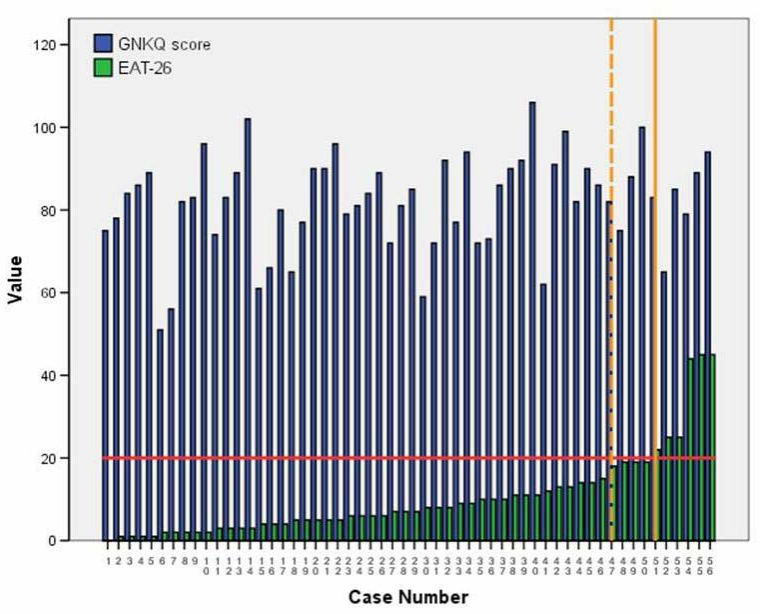
Distribution of the General Nutritional Knowledge Questionnaire score over the Eating Attitude Test (EAT-26) scores. Horizontal line represents the 'at risk' cutoff point. Vertical lines separate individuals in the 'at risk', 'borderline' and 'not at risk' categories.

Figure 3 depict the EAT-scores and GNKQ scores for each individual athlete in the sample. A small group of athletes (n = 4) scored very close to the cutoff point (18 < EAT-26 > 20). The mean GNKQ score for this borderline group was 86.25 ± 10.60 compared to 82.50 ± 9.99 for the 'at risk' group and 81.46 ± 12.17 for those with EAT-26 < 17. The difference between the mean scores were not significant (Kuskal Wallis χ^2 ^= .305, p = .859). There was no statistically significant relationship between nutrition knowledge (GNKQ) and eating attitude (EAT-26) for athletes (*r *= .177, *p *= .188) or controls (*r *= .077, *p *= .680).

### Nutrition knowledge and 'at risk' symptoms

Contrary to the expectation from Zawila and colleagues [12], no differences in mean nutrition knowledge scores were present for those either classified as 'at risk' or 'not at risk' for any component of the Triad among athletes (82.00 ± 13.05 and 81.26 ± 11.163, respectively; *t *= -.224, *p *= .824) or controls (81.50 ± 13.026 and 75.38 ± 12.79, respectively, *U *= 39.50, *p *= .445). Further analysis showed no significant difference in nutritional scores attained for either group for disordered eating (*U *= 150.50, *p *= .891), menstrual dysfunction (*U *= 354.50, *p *= .967) or bone mineral density (*U *= 91.50, *p *= .245). Mean scores and standard deviations are displayed in Table 3, which also shows a sub-section breakdown of nutrition knowledge scores. In general, 'at risk' athletes achieved a lower score in each subsection than their 'not at risk' counterparts but the differences were notably small and none of them were statistically significant at the p < .05 level.

**Table 3:** Mean Scores and Standard Deviations (in parentheses) of the General Nutritional Knowledge Questionnaire

	EAT-26		Menstrual dysfunction		Bone mineral density	
	'At risk'	'No risk'	'At risk'	'No risk'	'At risk'	'No risk'

Expert opinion	8.50 (1.06)	9.06 (0.51)	8.93 (0.81)	8.81 (0.55)	9.00 (0.92)	8.74 (0.36)
Food group	57.30 (2.25)	55.59 (2.25)	56.83 (3.58)	55.48 (2.44)	58.83 (4.04)	53.18 (1.57)
Choosing foods	8.90 (0.71)	8.69 (0.34)	8.81 (1.54)	8.71 (0.37)	9.17 (0.61)	8.35 (0.24)
Health problems	10.40 (1.90)	10.79 (0.91)	11.00 (1.45)	10.32 (0.99)	11.00 (1.64)	10.32 (0.64)
Nutritional knowledge	82.50 (9.99)	81.46 (12.52)	81.59 (11.74)	81.40 (12.43)	87.80 (7.60)	80.87 (12.36)

No significant relationship was found between and EAT-26 scores of athletes (*r *= .158, *p *= .231) and controls (*r *= .058, *p *= .753) indicating that attitude towards eating is independent of nutrition knowledge. This result is congruent with the findings of Packman and Kirk [15], where no relationship was found between the level of nutrition knowledge and attitudes toward fat consumption.

In summary, symptomatic behaviour of the Female Athlete Triad is more prevalent amongst athletes than controls in the present study. The nutrition knowledge of female endurance athletes was significantly higher than that of their non-athlete counterparts but no significant differences were observed in the nutrition knowledge scores attained by those athletes classified 'at risk' compared to those classified 'not at risk'.

## Discussion

To date, only one study has examined the prevalence of the Triad components amongst the non-athletic population. This is surprising given that the ACSM position stand [2] clearly states that all women are 'at risk' of its development. Although the control population was included predominantly to assess nutrition knowledge, it is interesting to examine differences in Triad risk behaviour compared to the athletic sample. EAT-26 scores ≥ 20 were attained by 3.1% and 8.3% of controls and athletes respectively. Amongst the athletic sample, menstrual dysfunction was significantly higher (*p *< .05), however of the 21 (43.8%) who were considered to be 'at risk', only one was also considered as 'at risk' of either disordered eating or low bone mineral density.

Initially these results may be of little concern, however it is important to consider that the Triad occurs on a continuum. Bearing in mind the detrimental physiological effects induced by the occurrence of any one component, a respondent is clearly putting themselves at increased risk of developing other aspects of the Triad. For this reason, Torstveit and Sungot-Borgen [26] classified those 'at risk' as respondents meeting any one of the criteria. This results in 42.4% of athletes and 12.5% of controls in the present study being 'at risk' of the Female Athlete Triad based on proxy measures for the three components. This is in keeping with studies conducted by Torstveit and Sungot-Borgen [26] who classified 60.4% of athletes 'at risk' of the triad, including the BMI < 18.5 criterion.

Results of this study show significantly higher nutrition knowledge amongst athletes compared to the normal population. Following basic guidelines for healthy eating is the most important dietary consideration for elite athletes [27]. The questionnaire covered these guidelines thus elevated scores imply better understanding of dietary needs and consequently improved eating behaviour. However, overall results from this study do not indicate this. 'At risk' EAT-26 scores were present in 10.2% of athletes (controls = 3.1%) and 16.7% had previously been diagnosed with either anorexia nervosa or bulimia nervosa compared to 3.1% of controls. This conclusion supports findings of previous research showing that athletes may know what the advisable behaviour is regarding eating and nutrition but tend not to follow these guidelines if it was not practical [28]. Studies regarding the effectiveness of nutrition education showed that while improvement in knowledge occurred, there was no difference observed in eating behaviour [25,29].

The reasons underlying the disordered eating despite the high level of nutrition knowledge may be both cognitive and motivational. People may have *inert knowledge*, which can be cited or recalled on a test but not applied to problems [30] or behavioural decisions. Alternatively, information may be available but consciously ignored or overwritten by reasons with higher priority (i.e. keeping weight unreasonably low for aesthetic or performance reasons). Individuals may possess the relevant information but they only use what is important to them [14].

Having the knowledge of health recommendations but not followed can be considered a form of risk taking [31]. Cook and Bellis showed that knowledge of health risks and risk-taking behaviour were peculiarly related: those with precise risk assessment were high risk takers whilst those who repeatedly over-estimated the risks exhibited low level of risk-taking behaviour [32]. Better than average nutrition knowledge does not necessarily have a positive effect on individual health. Athletes with heightened awareness may engage in risk taking behaviour by making excessive efforts to reduce calorie intake in order to stay lean, with negative consequences on performance and ultimately on health. Athletes may justify their unhealthy eating habits as being controlled, temporal and goal oriented behaviour. In a sporting arena where leanness often equates to success, daily decisions about what and how much to eat are a constant challenge to the female athlete. This phenomenon can be explained by the perceived sense of control over the risks. For example, decision in a simulated situation (i.e. driving), people with control (drivers) were more comfortable taking high level of risk than those who had no control (passengers) [33]. Additionally, in case of deliberate acts, motives for a given behaviour exert influence on the perceived control over the behaviour [34] and risks taken. The deliberately low daily energy intake (cognitive dietary restraint) is also likely to be reinforced by the subculture where low body weight is desirable and restricted eating is the perceived norm. Further research is needed to investigate the applicability of these explanations of the seemingly deliberate unhealthy dieting observed among female athletes.

Decisions about whether to engage in risky behaviour, e.g. restrictive eating, and the subsequent impact on health can be serious. Although some dispute the seriousness of the Triad [35,36], it is possible that this underestimation of the cumulative effects of one's behaviour is relevant to the Female Athlete Triad. Athletes scored significantly higher than controls in all nutrition knowledge topic areas, yet no relationship was observed between higher nutrition knowledge and decreased EAT-26 scores or vice versa. This suggests that 'at risk' taking behaviour, i.e. cognitive dietary restraint, is present.

The majority of female endurance athletes (88%) are consuming less than the minimum amount of energy recommended when training (45 kcal/kg/day) [25]. This may represent a chronic, low level stressor instigating cortisol release. High cortisol levels have been associated with reproductive disturbances and are known to have a direct effect on bone mineral density [37]. Numerous studies have shown that these sub-clinical disorders occur more frequently in women with high levels of cognitive dietary restrain [38-43] indicating that nutrition intervention programmes should focus on behavioural and psychosocial changes alongside nutritional awareness, particularly as disordered eating patterns, once established, are difficult to relinquish [14].

### Limitations

This study is considered explorative for a number of reasons. A large percentage of respondents were self-selected. Those with experience of the Triad disorders or a particular interest in nutrition or health issues may be more inclined to respond resulting in a known volunteer effect. Self-selection also meant the standard of athletes was not as 'elite' as desired. Even though criteria were set in order to filter out the 'recreational athlete', it was concluded that a broad range of abilities was included in the athletic sample.

Identification of 'at risk' factors is essential in the evaluation of the Triad [26]. It is therefore important to stress that this study examined 'at risk' behaviour of the Triad rather than the occurrence of the disorders themselves. To achieve this, cut-off points were designated for each component, thus borderline respondents may have been categorised incorrectly. However, because of the assessment criteria in each element of the Triad, such a 'close miss' could only happen regarding the disordered eating assessment, where the measurement was taken on a quasi-continuous scale (see Figure 3). Further research involving clinical interviews and dual energy x-ray absorptiometry (DXA) is required to assess the existence of one or more elements of the Triad accurately. Energy intake and expenditure should also be calculated and taken into account.

### Suggestions for future research

A number of studies have reported an inverse relationship between CDR and either menstrual dysfunction or low bone mineral density [36,37]. To date, no research has examined the direct relationship of CDR with the occurrence of disordered eating among athletes. Thus, to extend the work of this study, future research should focus on CDR measurement to identify potentially serious problems and consequences associated with poor nutrition choices despite good nutritional awareness. Food diaries, clinical assessment and interviews of those considered 'at risk' would provide a useful insight to the athlete's reasoning for dietary behaviour or restraint. Future studies should incorporate other potentially important factors, such as genetics, desired weight change and perceived pressure to lose weight, perceived health risk and predisposition to risk taking. Special attention should be given to athletes' participation in sports where leanness is considered advantageous.

## Conclusion

Our findings have applied implications. Although no direct evidence presented in our data indicates what factors are accountable for the higher percentage of athletes symptomatic of the Female Athlete Triad (e.g. risk taking behaviour such as cognitive dietary restraint), it was apparent that nutritional knowledge does not provide a compelling explanation for the 'at risk' status. Further research is required into determinants of disordered eating among certain athlete groups and findings of this study suggest that it is necessary to look beyond nutrition knowledge. The importance of developing a better understanding of deliberate restrictive dieting is underscored by the fact that this phenomenon is also observable in the young female non-athlete population.

In terms of intervention, if optimising performance is the dominant factor in motivating the female athlete, implementation of sound nutritional practices must be put in place. This requires a holistic approach, whereby the athlete's eating and lifestyle patterns and psychosocial influences are addressed. Education about the Triad as a disorder in its own right is necessary so athletes understand the consequences of their eating habits but simply providing or acquiring nutrition knowledge is not adequate to ensure that correct practice is performed. Use of nutritional supplements as a preventive measure should be considered for athletes who are at risk of prolonged negative energy balance.

## List of abbreviations

B.E.S.T.: Better Eating Safer Training Research Study. Note: This is a series of research projects of the The Orthopedic Specialty Hospital (Intermountain Healtcare, Utah, USA) investigating unhealthy behaviour and practices among high school athletes. One of the projects ('Eating, Sports and Health in Females') aims at assessing risk factors of the Female Athlete Triad.

BMD: Bone Mineral Density

CDR: Cognitive Dietary Restraint

DXA: dual energy x-ray absorptiometry. Note: in the literature, both 'DXA' and 'DEXA' are used to abbreviate the technique. In this paper, we used 'DXA'

EAT-26: Eating Attitude Test – 26

EAT-40: Eating Attitude test – 40

GNKQ: General Nutritional Knowledge Questionnaire

## Competing interests

The author(s) declare that they have no competing interests.

## Authors' contributions

PR-B conceived and designed the study, collected data and drafted the manuscript; AP contributed to the concept and design, analyses the data and drafted the manuscript; EQ contributed to the interpretation of the results and drafted the manuscript. All authors read and approved the final manuscript.
